# *ABCA1* rs1883025 and *CYP4F2* rs2108622 Gene Polymorphism Association with Age-Related Macular Degeneration and Anti-VEGF Treatment

**DOI:** 10.3390/medicina57090974

**Published:** 2021-09-16

**Authors:** Ruta Mockute, Alvita Vilkeviciute, Vilma Jurate Balciuniene, Reda Zemaitiene, Rasa Liutkeviciene

**Affiliations:** 1 Department of Ophthalmology, Lithuanian University of Health Sciences, Medical Academy, Eiveniu 2, LT-50009 Kaunas, Lithuania; jurate.balciuniene@kaunoklinikos.lt (V.J.B.); reda.zemaitiene@lsmuni.lt (R.Z.); rasa.liutkeviciene@lsmuni.lt (R.L.); 2 Neuroscience Institute, Lithuanian University of Health Sciences, Medical Academy, Eiveniu 2, LT-50009 Kaunas, Lithuania; alvita.vilkeviciute@lsmuni.lt

**Keywords:** age-related macular degeneration, rs2108622, rs1883025, gene polymorphisms, allele, retinal diseases

## Abstract

*Background and Objectives*: The age-related macular degeneration (AMD) pathophysiology is multifactorial, as it consists of interactions between aging, genetic, and environmental factors. We aimed to determine a relationship between AMD and the genes controlling lipid metabolism, and to assess its association with treatment results. The purpose was to find the *ABCA1* rs1883025 and *CYP4F2* rs2108622 gene polymorphisms in patients with exudative AMD (eAMD) treated with anti-VEGF. *Materials and Methods*: The study enroled 104 patients with eAMD and 201 healthy persons in a control group. The genotyping of rs1883025 and rs2108622 was performed using the RT-PCR method. The best-corrected visual acuity (BCVA) and central retinal thickness (CRT) were measured before anti-VEGF therapy, then at three and six months during the therapy, using optical coherence tomography (OCT). The patients were grouped to responders and non-responders according to the changes in BCVA and CRT. *Results*: The T allele at rs1883025 was more frequent in non-responder eAMD patients compared to responder eAMD patients (41.7% vs. 21.1%; *p* = 0.009). The analysis of rs2108622 gene polymorphism did not reveal any differences in the distribution of C/C, C/T, and T/T genotypes between the eAMD group and the control group (56.35%, 39.78%, and 3.87% in the eAMD group and 53.33%, 39.05% and 7.62% in the control group, respectively, *p* = 0.286). The comparison of CRT and BCVA between the rs2108622 genotypes revealed statistically significant differences: CRT was thicker for the CC carriers than for those with CT and TT genotypes (*p* = 0.030). *Conclusion*: The rs1883025 T allele was found to play a more significant role in non-responder eAMD patients compared to responder eAMD patients. The rs2108622 genotypes revealed statistically significant differences: CRT was thicker for the CC carriers than for those with CT and TT genotypes.

## 1. Introduction

AMD is the main cause of blindness in the developed countries [1,2]. Prevalence of this disease increases rapidly with the age over 75 years. As the population is aging, AMD is becoming a more and more serious problem in the world. Systematic reviews and meta-analysis have shown that 8.7% of the population have AMD, and the expected numbers will have reached 300 million by 2040 [3]. The complex etiology of AMD is associated with genetic and environmental risk factors [4]. There are two types of late AMD, i.e., atrophic and exudative. Atrophic AMD’s clinical signs are the presence of drusen and geographic atrophy of the retina. An exudative AMD’s signs are the invasion of abnormal choroidal membranes by newly formed blood vessels with the leakage of fluid under the retina [5]. Exudative AMD accounts for 80% to more than 90% of severe vision loss [2,5] and 80% of significant visual loss related to AMD [2]. Exudative AMD’s (eAMD) distinctive feature is choroidal neovascularization (CNV). It is an abnormal blood vessel growth from the choroid through the Bruch’s membrane [6]. Vascular endothelial growth factor (VEGF) is the main mediator in pathogenesis of CNV. It induces angiogenesis and increases vascular permeability [7]. Consequently, anti-vascular endothelial growth factor agents (anti-VEGF) have been the pole of the therapy for eAMD in the last decade [7]. Three anti-VEGF agents, pegaptanib, ranizumab, and bevacizumab, effectively treat eAMD [8,9]. With ageing, deposition of lipid particles in normal Bruch’s membrane leads to the creation of a lipid wall external to the basal lamina of RPE, aggravating nutrient exchange between the choriocapillaris and the RPE and partially destroying retinal function [10,11,12,13]. Lipids make up 40% of drusen [11,14,15]. Oxidized cholesterol is involved in AMD; its deposits have been found at BrM, choriocapillaris, and endothelial cell surfaces [16]. It also induces vascular endothelial growth factor production in RPE cell cultures and can cause choroidal neovascularization in AMD’s wet form [16]. Cytochrome P450 (CYP) enzymes belong to a superfamily of oxidoreductases that catalyse the metabolism of both endogenous substances and xenobiotics, including drugs [17]. Metabolites generated in the CYP epoxygenase pathway are related to inflammation and angiogenesis. It is known that the CYP-derived lipid metabolites 17,18-epoxyeicosatetraenoic acid and 19,20-epoxydocosapentaenoic acid play a vital role in alleviating choroidal neovascularization (CNV) severity by regulating leukocyte recruitment and the inflammatory microenvironment in CNV lesions [18]. *CYP4F2* is a member of the CYP450 superfamily and plays the main role in the metabolism of exogenous and endogenous compounds [19]. The liver, heart, lungs, and kidneys are expressing *CYP4F2*, which is involved in leukotriene B4 (LTB4) and 20-hydroxyarachidonic acid (20-HETE) metabolism [20]. There are known associations of AMD with several genes implicated in high density lipoprotein (HDL) metabolism [21]. Genome-wide association (GWAS) reported associations of AMD with the *ATP-binding cassette transporter A1* (*ABCA1*) gene [22]. ABCA1 is a sterol-induced membrane protein that mediates the excess cholesterol transfer from cells to lipid-poor apolipoprotein (apo)A-I, the major protein component of HDLs [23]. Mutations in human *ABCA1* are associated with a severe HDL deficiency, cholesterol deposition in tissue macrophages, and prevalent cardiovascular disease [24]. RPE cells express apolipoprotein ATP binding cassette transporter A1 (*ABCA1*) [25]. Reverse cholesterol transport mediated through *ABCA1* releases cholesterol to apoE and apoA1, forming high-density lipoproteins to recycle lipids, including docosahexaenoic acid (DHA) and cholesterol [26].

We have conducted research to find the association between eAMD and the genes *ABCA1* rs1883025 and *CYP4F2* rs2108622, taking part in lipid metabolism, and to determine their association with Anti-VEGF treatment.

## 2. Materials and Methods

The study was conducted at the Ophthalmology Laboratory, Neuroscience Institute, Lithuanian University of Health Sciences (LUHS), (Number-BE-2-/48). The study sample was comprised of patients with eAMD group, *n* = 104, and a control group included 201 subjects.

### 2.1. Control Group Formation

The control group consisted of 201 subjects who had no ophthalmologic pathology on examination and agreed to participate in this study. The control group was constructed of 134 women and 67 men aged from 67 to 94 years.

The differences of gender and age between the groups were not statistically significant (Table 1).

### 2.2. Ophthalmological Evaluation

Best-corrected visual acuity (BCVA) was measured using the standard procedure adapted for the Age-Related Eye Disease Study (AREDS) at a 5-m distance from the chart (letters on the ETDRS logMAR chart); for subjects with significantly impaired vision, at one meter. During examination, intraocular pressure was measured as well. Pupils were dilated with tropicamide 1%, after which fundoscopy, using slit-lamp biomicroscopy with a double aspheric lens of +78 dioptre, was performed. Two ophthalmologists diagnosed AMD.

CRT measurements were performed before therapy, at three months and at six months after the first anti-VEGF intravitreal injection using optical coherence tomography (Nidek RS 3000). All patients, older than 50 years, with the presence of exudative or haemorrhagic features in the macula, had no previous intravitreal anti-VEGF treatment, and had a follow-up at least six months after the first injection of anti-VEGF.

The following subject inclusion criteria were used: (i) All gender patients, older than 50 years, with the presence of exudative or haemorrhagic features in the macula; (ii) The diagnosis of AMD was confirmed by two ophthalmologists by performing OCT; (iii) an informed person consent form has been signed.

The following subject exclusion criteria were used: (i) unrelated eye disorders, e.g., high refractive error, cloudy cornea, lens opacity (nuclear, cortical, or posterior subcapsular cataract) except minor opacities, keratitis, acute or chronic uveitis, glaucoma, or diseases of the optic nerve; (ii) systemic illnesses, e.g., diabetes mellitus, malignant tumors, systemic connective tissue disorders, chronic infectious diseases, or conditions following organ or tissue transplantation; (iii) ungraded color fundus photographs resulting from obscuration of the ocular optic system or because of fundus photograph quality.

Based on OCT and BCVA clinical data, the patients were divided into two groups: responders and non-responders. BVCA was evaluated before therapy, at three months and at six months after the first anti-VEGF intravitreal injection. Impairment of BCVA was considered when patients loss one or more signs from the chart. Insufficient effect in structural changes was considered when according to the OCT there was an increase in CRT of 100 μM after six months from the first injection, or persistent macular oedema.

The non-responder group was defined as patients who had two or more signs of these: reduction in BCVA, an increase in CRT of 100 μM after six months from the first injection, compared to the baseline, and persistent macular oedema. The responder group was defined as patients who had less than two signs.

### 2.3. DNA Extraction and Genotyping

DNA extraction and genotyping was described in our previous research [27,28]. The DNA extraction and analysis of *ABCA1* (rs1883025) and *CYP4F2* (rs2108622) polymorphisms were carried out at the laboratory Ophthalmology at the Institute of Neuroscience of LUHS. DNA was extracted from 200 μL venous blood (white blood cells) using a DNA purification kit based on the silica-based membrane technology utilizing a genomic DNA extraction kit (GeneJET Genomic DNA Purification Kit, K072, Thermo Fisher Scientific Inc., Vilnius, Lithuania), according to the manufacturer’s recommendations.

The genotyping of single-nucleotide polymorphisms (SNPs) of *ABCA1* and *CYP4F2* were determined using TaqMan^®^ Genotyping assays (Thermo Fisher Scientific Inc., Foster city, CA, USA): rs1883025; assay ID C___2959486_10) and rs2108622; assay ID C__16179493_40 was carried out using the real-time polymerase chain reaction (RT-PCR) method.

Appropriate real-time PCR mixtures were prepared for determining selected SNPs according to the manufacturer’s recommendations.

A PCR reaction mixture (9 μL) was poured into the 72 wells of the Rotor-Disc, and then 1 μL of matrix DNA of the samples (~10 ng) and 1 μL of a negative control (−K) were added.

The genotyping was performed using a Rotor-Gene Q real-time PCR quantification system (Qiagen, Hilden, Germany). The Allelic Discrimination program was used during the real-time PCR. Then, the assay was continued following the manufacturer’s manual (www.qiagen.com, Rotor-Gene Q User Manual, accessed on 7 July 2021). After that, the Allelic Discrimination program was completed, and the genotyping results were received. The program determined the individual genotypes according to different detectors’ fluorescence intensity rate (VIC and FAM dyes).

### 2.4. Statistical Analysis

The SPSS/W 20.0 software (Statistical Package for the Social Sciences for Windows, Inc., Chicago, IL, USA) was used for statistical analysis. The data are presented as absolute numbers with percentages in brackets, median values, and minimum (min.) and maximum (max.) or interquartile range (IQR).

Hardy-Weinberg analysis was performed to compare the observed and expected frequencies of rs1883025 and rs2108622 using the χ^2^ test in all groups. The distribution of the rs1883025 and rs2108622 single-nucleotide polymorphism (SNP) in the AMD and control groups was compared using the χ^2^ test or the Fisher exact test.

Differences were considered statistically significant when *p* < 0.05.

## 3. Results

A total of 104 patients with eAMD were included in the study according to the subject inclusion and exclusion criteria. The control group was formed of 201 persons. There were 66.7% (*n* = 134) of women in the control group and 68.3% (*n* = 71) in the eAMD group.

The genotyping rs1883025 was performed in patients with eAMD and the control group subjects. The results of rs2108622 from our earlier study are presented in Table 2 [27] and results of rs1883025 are presented in Table 3. The distribution of the analyzed rs2108622 and rs1883025 genotypes and allele frequencies in patients with eAMD and the control group subjects matched the Hardy-Weinberg equilibrium. SNPs analysis in the overall study group did not reveal any differences in the genotype distributions between patients with eAMD and control group subjects.

Based on their response to treatment, 104 patients with eAMD were classified into responder (*n* = 86) and non-responder groups (*n* = 18).

Comparison of the rs1883025 genotype frequency between the responder and non-responder groups did not show statistically significant differences, but the T allele was statistically significantly more frequent in the non-responder than in the responder group (41.7% vs. 21.1%; *p* = 0.009).

Comparing the rs2108622 genotype distributions between the responder and non-responder groups did not show statistically significant differences (Table 4 and Table 5).

The comparison between the responder and non-responder group is shown in Table 6. The mean CRT was not significantly different between the two groups (*p* > 0.05), though the mean BCVA at baseline was statistically significantly worse in the responder group than in non-responder (0.30 (0.25) vs. 0.40 (0.48), *p* = 0.028). However, at six months after the first injection, an improved mean BCVA compared to the corresponding baseline measurement was observed in the responder group, while in the non-responder group the mean BCVA after six months was worse (0.35 (0.28) vs. 0.3 (0.38), respectively; *p* = 0.091) (Table 6).

The mean BCVA, obtained at baseline and at three, six, and twelve months from the first intravitreal injection of anti-VEGF, and CRT, obtained at baseline and after three and six months, were compared between the *ABCA1* and CYP450 genotypes. Besides, changes in BCVA and CRT measurements at observed time points were evaluated according to the genotypes.

The comparison of CRT and BCVA between the rs1883025 genotypes did not show statistically significant differences (Table 7).

The comparison between the rs2108622 genotypes revealed statistically significant differences: CRT was thicker for the CC carriers than for those with CT and TT genotypes (*p* = 0.030) (Table 8).

## 4. Discussion

Our study investigated *ABCA1* rs1883025 and *CYP450* rs2108622 polymorphisms in eAMD patients treated with anti-VEGF injections. In this prospective study, we appreciated BCVA, and also investigated structural changes in the retina by measuring CRT before and after treatment. We investigated the association of *ABCA1* rs1883025 and *CYP4F2* rs2108622 with the improvement of VA in response to eAMD treatment, from both a functional (BCVA) and structural (CRT) aspect.

After thorough statistical analysis, we found statistically significant differences in *ABCA1* rs1883025 allele distribution between the responder and non-responder groups. The rs1883025 T allele was more frequent in the non-responder group than in the responder group (41.7% vs. 21.1%, respectively; *p* = 0.009). However, there were no statistically significant differences of structural and functional changes between the rs1883025 genotypes.

The comparison of CRT and BCVA between the rs2108622 genotypes revealed statistically significant differences: CRT was thicker for the CC carriers than for those with CT and TT genotypes (*p* = 0.030). Comparing the rs2108622 genotypes and alleles between the responder and non-responder groups we did not find statistically significant differences.

Novelty of our study is the investigation of the associations between SNPs in eAMD patients and eAMD treatment based on structural and functional changes. There are almost no studies including all of these factors and no studies with these gene polymorphisms. We compared our data with similar studies. Many genetic variants in genes regulating lipid metabolism and transfer among lipoproteins play a role in AMD development [29,30]. Retinal pigment epithelial (RPE) cells express components that are necessary for lipoprotein ingestion, cholesterol synthesis, lipoprotein synthesis, and secretion, as well as reverse cholesterol transport (RCT) [11,31,32]. Also, ocular production of lipoproteins is the primary source of lipoproteins found in drusen [33]. Drusen consist of polar lipids, such as un-esterified (free) cholesterol (UC) and phosphatidylcholine (PC), neutral lipids, such as cholesteryl esters (CEs), and several lipid-binding proteins (apolipoproteins) [15].

Fritsche LG et al. have analyzed many genes association with AMD and found that several genes, which are involved in the generation and remodelling of high-density lipoproteins (HDLs), namely ATP-binding cassette transporter A1 (*ABCA1*), apolipoprotein E (*APOE*), cholesteryl ester transfer protein (*CETP*), and hepatic lipase C (*LIPC*), are also linked to AMD [30].

In this context, one of genes we studied was *ABCA1*. It encodes a transporter which transports lipids through the membrane. That generates HDLs together with its partner ABCG1. Both transporters use ATP to flip lipids, mainly UC and phospholipids (PLs), but also sphingomyelins (SMs) and oxysterols, from the inner leaflet of the plasma membrane to extracellular lipophilic acceptors such as apolipoproteins or nascent HDLs [34]. Federica Storti et al. have investigated mouse RPE survival in vivo and determined a relevant part of the *ABCA1*/ABCG1 lipid efflux pathway, suggesting that the pathology of AMD is related with an impaired lipid metabolism via *ABCA1* [35].

Yu Y et al. have evaluated fundus photography of 3066 subjects, including control group (221), intermediate drusen group (814), large drusen group (949) and patients with advanced AMD (1082). They have found that *LIPC* and *ABCA1* genes are associated with intermediate, large drusen, and advanced AMD development [22]. Furthermore, Fanging et al. found that rs1883025 T allele was significantly related with development of AMD [36]. Some studies did not confirm these findings, but it could be based on ethnic differences [37].

*CYP4F2* rs2108622 also linked to AMD. The *CYP4F2* protein belongs to the P450 superfamily and is associated with the production of 20-HETE thus influencing endothelial dysfunction, vascular oxidative stress, and high peripheral vascular resistance [38]. As found in the earlier study, the *CYP4F* (1347C>T) *T/T* genotype was more frequent in males with eAMD compared to healthy males’ group (10.2% vs. 0.8%; *p* = 0.0052). Therefore *CYP4F2* (1347C>T) *T*/*T* genotype was less frequent in eAMD females compared to healthy females (0.8% vs. 6.2%; *p* = 0.027) [27].

There are some studies similar to our present study. Kubicka-Trzaska A. et al. enrolled 106 patients with AMD and analyzed the correlation between Complement proteins genes (Y402H, E318D, and R102G) polymorphisms response to intravitreal anti-VEGF treatment in AMD patients. They have found that the *CFH* rs1061170 CC genotype may be related with AMD and negative response to anti-VEGF treatment [39]. Also, a meta-analysis showed that Y402H (rs1061170) might be related with neovascular AMD treatment response (especially to the anti-VEGF agents) [40]. Guohai et al. have done meta-analysis and confirmed the role of *CFH* Y402H in AMD treatment for Caucasians. For the *CFH* Y402H polymorphism, anti-VEGF treatment was much less effective in AMD patients with the *CFH* CC genotype [41].

Sanjeewa et al. research has shown that the *APOE* ε4 allele’s presence conferred significantly better visual functions after anti-VEGF treatment than did the ε2 allele [42]. Another study has shown that *IL1RL1* rs1041973 might play a protective role against macular thickening in eAMD patients [28].

On the other hand, there are meta-analyses where genetic factors do not predict treatment efficiency. *HTRA1* rs11200638 polymorphism was not associated with anti-VEGF treatment and eAMD better outcomes [43]. These findings suggest a personalized approach of anti-VEGF treatment for each patient based on their genetic results.

The study’s main advantage is that there are no other studies analysing associations between eAMD treatment and genes in the Baltic countries. Another advantage is the study structure: we divided patients into groups by treatment effectiveness based on functional and morphological changes.

In our opinion, the study should be replicated with a larger number of eAMD patients and controls. Furthermore, the subjects should be further examined and the extended-term visual function between their genotypes should be compared.

## 5. Conclusions

In conclusion, we found that the rs1883025 T allele plays a more significant role in non-responder eAMD patients compared to responder eAMD patients. The comparison of CRT and BCVA between the rs2108622 genotypes revealed statistically significant differences: CRT was thicker for the CC carriers than for those with CT and TT genotypes.

## Figures and Tables

**Table 1:** Demographic data of the study population.

	Group		*p* Value
	Exuxudative AMD (eAMD) *n* = 104	Control *n* = 201	
Men, *n* (%)	33 (31.7%)	67 (33.3%)	0.777
Women, *n* (%)	71 (68.3%)	134 (66.7%)	
Age median (min; max)	77 (61; 92)	75 (67; 94)	0.186

*p* value—significance level.

**Table 2:** Frequency of the *CYP4F2* rs2108622 genotypes in the eAMD and control groups.

Genotype/ Allele	Frequency (%)				
	Control Group *n* (%) (*n* = 210)	*p* Value HWE	eAMD Group *n* (%) (*n* = 181)	*p* Value HWE	*p* Value
Genotype		0.854		0.187	
C/C	112 (53.33)		102 (56.35)		χ^2^= 2.501 *p* = 0.286
T/C	82 (39.05)		72 (39.78)		
T/T	16 (7.62)		7 (3.87)		
Total	210 (100)		181 (100)		
Allele					
C	336 (74.67)		276 (76.25)		χ^2^= 0.269 *p* = 0.604
T	114 (25.33)		86 (23.75)		

*p* value—significance level (*alfa* = 0.05), *p* value HWE—significance level (*alfa* = 0.05) by Hardy-Weinberg equilibrium.

**Table 3:** Frequency of the *ABCA1* rs1883025 genotypes in the eAMD and control groups.

Genotype/ Allele	Frequency (%)				
	Control Group *n* (%) (*n* = 210)	*p* Value HWE	eAMD Group *n* (%) (*n* = 181)	*p* Value HWE	*p* Value
Genotype		0.436		0.961	
C/C	125 (62.2)		59 (56.7)		χ^2^ = 1.848 *p* = 0.397
T/C	63 (31.3)		34 (32.7)		
T/T	13 (6.5)		11 (2.6)		
Total	201 (100)		104 (100)		
Allele					
C	313 (77.9)		152 (73.1)		χ^2^ = 1.731 *p* = 0.188
T	89 (22.1)		56 (26.9)		

*p* value—significance level (*alfa* = 0.05), *p* value HWE—significance level (*alfa* = 0.05) by Hardy-Weinberg equilibrium.

**Table 4:** Frequency of the *ABCA1* rs1883025 genotypes in the responder and non-responder groups.

Genotype/ Allele	Frequency (%)		
	Responders *n*(%) (*n* = 86)	Non-Responders *n* (%) (*n* = 18)	*p* Value
Genotype			
C/C	53 (61.6)	6 (33.3)	χ^2^ = 4.858 *p* = 0.088
T/C	25 (29.1)	9 (50)	
T/T	5 (9.3)	3 (16.7)	
Total	86 (100)	18 (100)	
Allele			
C	131 (78.9)	21 (58.3)	χ^2^ = 6.729 *p* = 0.009
T	35 (21.1)	15 (41.7)	

*p* value—significance level (*alfa* = 0.05).

**Table 5:** Frequency of the *CYP4F2* rs2108622 genotypes in the responder and non-responder groups.

Genotype/ Allele	Frequency (%)		
	Responders *n*(%) (*n* = 86)	Non-Responders *n* (%) (*n* = 18)	*p* Value
Genotype			
C/C	50 (58.1)	11 (61.1)	χ^2^ = 0.55 *p* = 0.973
T/C	31 (36)	6 (33.3)	
T/T	5 (5.8)	1 (5.6)	
Total	86 (100)	18 (100)	
Allele			
C	131 (76.2)	28 (77.8)	χ^2^ = 0.043 *p* = 0.836
T	41 (23.8)	8 (22.2)	

*p* value—significance level (*alfa* = 0.05).

**Table 6:** Central retinal thickness (CRT)r and BCVA comparison between the responder and non-responder groups.

Parameter	Time Point	Responders Group	Non-Responders Group	*p*-Value
Mean Central retinal thickness (CRT) (μM) Median (interquartile range)	Baseline	338 (100)	296 (105)	0.109
	Three months	276 (98)	307 (110)	0.299
	Six months	275 (88)	335 (105,5)	0.360
Mean BCVA Median (interquartile range)	Baseline	0.30 (0.25)	0.40 (0.48)	0.028
	Three months	0.35 (0.3)	0.40 (0.47)	0.557
	Six months	0.35 (0.28)	0.30 (0.38)	0.091

*p* value—significance level (*alfa* = 0.05).

**Table 7:** CRT and BCVA associations with *ABCA1* rs1883025 genotypes.

Parameter	Time Point	TT + TC	CC	*p* Value
Mean CRT (μM) Median (interquartile range)	Baseline	313 (87)	350 (102.5)	0.171
	Three months	269 (110)	280 (102)	0.099
	Six months	271 (111)	282 (106)	0.190
BCVA Median (interquartile range)	Baseline	0.32 (0.34)	0.3 (0.24)	0.188
	Three months	0.35 (0.4)	0.4 (0.3)	0.872
	Six months	0.35 (0.4)	0.3 (0.3)	0.744
Changes in BCVA Median (interquartile range)	Six months	0.00 (0.2)	0.05 (0.1)	0.081
	12 months	0.00 (0.26)	0.08 (0.2)	0.102
Changes in CRT Median (interquartile range)	Six months	27 (72.5)	15 (94)	0.691

*p* value—significance level (*alfa* = 0.05).

**Table 8:** CRT and BCVA associations with *CYP4F2* rs2108622 genotypes.

Parameter	Time Point	TT + TC	CC	*p* Value
Mean central retinal thickness (μM) Median (interquartile range)	Baseline	307.5 (84.5)	350 (94.25)	0.094
	Three months	265 (76.5)	280 (114.25)	0.092
	Six months	271 (72.25)	291.5 (117.75)	0.030
BCVA Median (interquartile range)	Baseline	0.265 (0.24)	0.3 (0.21)	0.952
	Three months	0.325 (0.3)	0.4 (0.4)	0.834
	Six months	0.3 (0.27)	0.31 (0.41)	0.732
Changes in BCVA Median (interquartile range)	Six months	0.035 (0.1)	0.00 (0.19)	0.261
	12 months	0.085 (0.18)	0.035 (0.28)	0.698
Changes in central retinal thickness Median (interquartile range)	Six months	49 (103.5)	24 (99)	0.410

*p* value—significance level (*alfa* = 0.05).

## Data Availability

All data could be seen in this manuscript.

## References

[1] Jager R.D., Mieler W.F., Miller J.W. (2008). Age-related macular degeneration. N. Engl. J. Med..

[2] Congdon N., O’Colmain B., Klaver C.C. (2004). Causes and prevalence of visual impairment among adults in the United States. Arch. Ophthalmol..

[3] Wong W.L., Su X., Li X., Cheung C.M.G., Klein R., Cheng C.Y., Wong T.Y. (2014). Global prevalence of age-related macular degeneration and disease burden projection for 2020 and 2040: A systematic review and meta-analysis. Lancet Glob. Health.

[4] García-Layana A., Cabrera-Lopez F., Garcia-Arumi J., Arias-Barquet L., Ruiz-Moreno J.M. (2017). Early and intermediate age-related macular degeneration: Update and clinical review. Clin. Interv. Aging.

[5] Friedman D.S., O’Colmain B.J., Munoz B., Tomany S.C., McCarty C., de Jong P.T., Nemesure B., Mitchell P., Kempen J. (2004). Eye Diseases Prevalence Research Group Prevalence of age-related macular degeneration in the United States. Arch. Ophthalmol..

[6] Gess A.J., Fung A.E., Rodriguez J.G. (2011). Imaging in neovascular age-related macular degeneration. Semin. Ophthalmol..

[7] Martin D.F., Maguire M.G., Fine S.L. (2012). Ranibizumab and bevacizumab for treatment of neovascular age-related macular degeneration: Two-year results. Ophthalmology.

[8] Gragoudas E.S., Adamis A.P., Cunningham E.T., Feinsod M., Guyer D.R. (2004). VEGF inhibition study in ocular neovascularization clinical trial group, pegaptanib for neovascular age-related macular degeneration. N. Engl. J. Med..

[9] Ferrara N., Damico L., Shams N., Lowman H., Kim R. (2006). Development of ranibizumab, an anti-vascular endothelial growth factor antigen binding fragment, as therapy for neovascular age-related macular degeneration. Retina.

[10] Bretillon L., Acar N., Seeliger M.W., Santos M., Annick M., Juanéda P., Martine L., Grégoire S., Joffre C., Bron A.M. (2008). ApoB100, LDLR−/− mice exhibit reduced electroretinographic response and cholesteryl esters deposits in the retina. Investig. Ophthalmol. Vis. Sci..

[11] Curcio C.A., Johnson M., Rudolf M., Huang J.D. (2011). The oil spill in ageing Bruch membrane. Br. J. Ophthalmol..

[12] Fliesler S.J., Bretillon L. (2010). The ins and outs of cholesterol in the vertebrate retina. J. Lipid Res..

[13] Kishan A.U., Modjtahedi B.S., Martins E.N., Modjtahedi S.P., Morse L.S. (2011). Lipids and age-related macular degeneration. Surv. Ophthalmol..

[14] Curcio C.A., Johnson M., Huang J.D., Rudolf M. (2010). Apolipoprotein B-containing lipoproteins in retinal aging and age-related macular degeneration. J. Lipid Res..

[15] Wang L., Clark M.E., Crossman D.K., Kojima K., Messinger J.D., Mobley J.A., Curcio C.A. (2010). Abundant lipid and protein components of drusen. PLoS ONE.

[16] Moreira E.F., Larrayoz I.M., Lee J.W., Rodríguez I.R. (2009). 7-Ketocholesterol is present in lipid deposits in the primate retina: Potential implication in the induction of VEGF and CNV formation. Investig. Ophthalmol. Vis. Sci..

[17] Rendic S., Guengerich F.P. (2015). Survey of Human Oxidoreductases and Cytochrome P450 Enzymes Involved in the Metabolism of Xenobiotic and Natural Chemicals. Chem. Res. Toxicol..

[18] Hasegawa E., Inafuku S., Mulki L., Okunuki Y., Yanai R., Smith K.E., Kim C.B., Klokman G., Bielenberg D.R., Puli N. (2017). Cytochrome P450 monooxygenase lipid metabolites are significant second messengers in the resolution of choroidal neovascularization. Proc. Natl. Acad. Sci. USA.

[19] Tatarunas V., Kupstyte N., Giedraitiene A., Skipskis V., Jakstas V., Zvikas V., Lesauskaite V. (2017). The impact of CYP2C19*2, CYP4F2*3, and clinical factors on platelet aggregation, CYP4F2 enzyme activity, and 20-hydroxyeicosatetraenoic acid concentration in patients treated with dual antiplatelet therapy. Blood Coagul. Fibrinolysis.

[20] Fava C., Ricci M., Melander O., Minuz P. (2012). Hypertension, cardiovascular risk and polymorphisms in genes controlling the cytochrome P450 pathway of arachidonic acid: A sex-specific relation?. Prostaglandins Other Lipid Mediat..

[21] Chen W., Stambolian D., Edwards A.O., Branham K.E., Othman M., Jakobsdottir J., Tosakulwong N., Pericak-Vance M.A., Campochiaro P.A., Klein M.L. (2010). Genetic variants near TIMP3 and high-density lipoprotein-associated loci influence susceptibility to age-related macular degeneration. Proc. Natl. Acad. Sci. USA.

[22] Yu Y., Reynolds R., Fagerness J., Rosner B., Daly M.J., Seddon J.M. (2011). Association of variants in the LIPC and *ABCA1* genes with intermediate and large drusen and advanced age-related macular degeneration. Investig. Ophthalmol. Vis. Sci..

[23] Oram J.F., Heinecke J.W. (2005). ATP-binding cassette transporter A1: A cell cholesterol exporter that protects against cardiovascular disease. Physiol. Rev..

[24] Singaraja R.R., Brunham L.R., Visscher H., Kastelein J.J., Hayden M.R. (2003). Efflux and atherosclerosis: The clinical and biochemical impact of variations in the ABCA1 gene. Arterioscler. Thromb. Vasc. Biol..

[25] Stewart J.M., Bailey K.R., Duncan K.G., Kane J.P., Schwartz D.M. (2003). Expression of reverse cholesterol transport protiens SR-BI, SR-BII and ABCA1 in the human retinal pigment epithelium. Ophthalmol. Vis. Sci..

[26] Tserentsoodol N., Gordiyenko N.V., Pascual I., Lee J.W., Fliesler S.J., Rodriguez I.R. (2006). Intraretinal lipid transport is dependent on high density lipoprotein-like particles and class B scavenger receptors. Mol. Vis..

[27] Sakiene R., Vilkeviciute A., Kriauciuniene L., Balciuniene V.J., Buteikiene D., Miniauskiene G., Liutkeviciene R. (2016). CYP4F2 (rs2108622) Gene Polymorphism Association with Age-Related Macular Degeneration. Adv. Med..

[28] Vilkeviciute A., Bastikaityte N., Mockute R., Cebatoriene D., Kriauciuniene L., Zemaitiene R., Liutkeviciene R. (2020). The Role of SNPs in IL1RL1 and IL1RAP Genes in Age-related Macular Degeneration Development and Treatment Efficacy. In Vivo.

[29] Grassmann F., Heid I.M., Weber B.H. (2017). International AMD Genomics Consortium (IAMDGC). Recombinant Haplotypes Narrow the ARMS2/HTRA1 Association Signal for Age-Related Macular Degeneration. Genetics.

[30] Fritsche L.G., Igl W., Bailey J.N., Grassmann F., Grassmann F., Sengupta S., Bragg-Gresham J.L., Burdon K.P., Hebbring S.J., Wen C. (2016). A large genome-wide association study of age-related macular degeneration highlights contributions of rare and common variants. Nat. Genet..

[31] Li C.M., Clark M.E., Chimento M.F., Curcio C.A. (2006). Apolipoprotein localization in isolated drusen and retinal apolipoprotein gene expression. Investig. Ophthalmol. Vis. Sci..

[32] Zheng W., Reem R.E., Omarova S., Huang S., DiPatre P.L., Charvet C.D., Curcio C.A., Pikuleva I.A. (2012). Spatial distribution of the pathways of cholesterol homeostasis in human retina. PLoS ONE.

[33] Dashti N., McGwin G., Owsley C., Curcio C.A. (2006). Plasma apolipoproteins and risk for age related maculopathy. Br. J. Ophthalmol..

[34] Li G., Gu H.M., Zhang D.W. (2013). ATP-binding cassette transporters and cholesterol translocation. IUBMB Life.

[35] Storti F., Klee K., Todorova V., Steiner R., Othman A., van der Velde-Visser S. (2019). Impaired ABCA1/ABCG1-mediated lipid efflux in the mouse retinal pigment epithelium (RPE) leads to retinal degeneration. eLife.

[36] Yafeng W., Mingxu W., Yue H., Rui Z., Le M. (2016). ABCA1 rs1883025 polymorphism and risk of age-related macular degeneration. Graefes Arch. Clin. Exp. Ophthalmol..

[37] Fanging L., Yingjie L., Mingwu L., Yaoyao S., Bai Y., Yang F., Guo J., Chen Y., Huang L., Li X. (2014). ABCA1 rs1883025 polymorphism shows no association with neovascular age-related macular degeneration or polypoidal choroidal vasculopathy in a Northern Chinese population. Ophthalmic. Res..

[38] Waldman M., Peterson S.J., Arad M., Hochhauser E. (2016). The role of 20-HETE in cardiovascular diseases and its risk factors. Prostaglandins Other Lipid Mediat..

[39] Kubicka T.A., Karska-Basta I., Kobylarz J., Dziedzina S., Sanak M., Romanowska-Dixon B. (2016). Association between Y402H, E318D and R102G polymorphisms of complement proteins genes and the response to intravitreal anti-VEGF treatment in patients with neovascular age-related macular degeneration. Klin. Oczna.

[40] Chen H., Yu K., Wu G.Z. (2012). Association between variant Y402H in age-related macular degeneration (AMD) susceptibility gene CFH and treatment response of AMD: A meta-analysis. PLoS ONE.

[41] Guohai C., Radouil T., Ensheng L., Fangzheng J., Mao S., Tong Y. (2015). Pharmacogenetics of Complement Factor H Y402H Polymorphism and Treatment of Neovascular AMD with Anti-VEGF Agents: A Meta-Analysis. Sci. Rep..

[42] Wickremasinghe S.S., Wie J., Lim J., Chauhan D.S., Robman L., Richardson A.J., Hageman G., Baird P.N., Guymer R. (2011). Variants in the APOE gene are associated with improved outcome after anti-VEGF treatment for neovascular AMD. Investig. Ophthalmol. Vis. Sci..

[43] Zhou Y.L., Chen C.L., Wang Y.X., Tong Y., Fang X.L., Li L., Wang Z.Y. (2017). Association between polymorphism rs11200638 in the HTRA1 gene and the response to anti-VEGF treatment of exudative AMD: A meta-analysis are current standard treatments. However, the outcome of anti-VEGF therapeutics is not uniform in all patients. BMC Ophthalmol..

